# Monthly Summary

**Published:** 1874-05

**Authors:** 


### MONTHLY SUMMARY.

---

*Apparent Death—Recognition by Faradization.*—Prof. Rosenthal, of Vienna, has recorded an interesting case of trance detected by faradization in a hysterical woman whose death had already been certified by a country practitioner. It had been found that a looking-glass held to the mouth of the woman did not show any moisture, and that melted sealing-wax dropped on the skin caused no reflex movements. Rosenthal, who was accidentally present, found the skin pale and cold, the pupils contracted and insensible to light, the upper and lower extremities relaxed, the heart's impulse and the radial pulse imperceptible. Auscultation, however, showed a feeble, dull, and intermittent sound in the cardiac region. No respiratory murmurs were audible. All the muscles of the face and the extremities responded well to the faradic current. Although the patient had been apparently dead for thirty-two hours, he thereupon informed the relations that she was only in a trance, and recommended that attempts at resuscitation should be perseveringly followed. On the following day he received a telegram saying that the woman awoke spontaneously twelve hours after his visit, and gradually recovered her speech and movements. Four months afterwards the patient called upon him, and informed him that she knew nothing of the commencement of the attack of lethargy in which she had been ; that she had afterwards heard the people about her talk of her death, but had been utterly unable to give the slightest sign of life. Two years afterwards she was still alive and tolerably well.—*British Medical Journal.*

---

*Death from Chloroform—Damages.*—It will be remembered some months ago a M. Caron took his wife to a dentist at Lille, who, in his presence, gave her chloroform for the purpose of painlessly extracting some teeth, but she died under the influence of the anæsthetic. The dentist was condemned to one month's imprisonmeut, and a fine of 500 francs (£20) for "homicide through imprudence;" on appeal he was relieved from the sentence of imprisonment. M. Caron, however, brought a further action in the civil court for damages in the name of himself and of his son, a minor. The judgment accepted as proved (from the sentences of the criminal court and the Court of Appeal) the fact of homicide through imprudence, and condemned the dentist to damages of 4,000 francs (£160) divided thus: 1,000 francs to M. Caron, and 3,000 francs to his young son, to be placed in the funds, the whole capital and interest to be handed to him on his majority. In this case Madam Caron had taken chloroform with impunity on a previous occasion, and the husband had by his presence fully authorized its administration on this occasion.—*London Med. Record.*

---

*Treatment of Chloroform Asphyxiation.*—Dr. Campbell, of Paris, recommends, in a late number of the *Journal de Therapeutique*, to place persons threatened with death from inhalations of chloroform head downwards and feet upwards for between ten and fifteen minutes. He considers that death arises through syncope due to cerebral anæmia; hence the advantage of inducing an artificial cerebral congestion. The usual efforts at mechanical breathing, excitement of respiratory nerves, the drawing out of the tongue, insufflation into the lungs, etc., may be had recourse to concurrently. Dr. Campbell mentions only one case where this method succeeded; it was suggested by Nélatou during an operation performed at Paris by Marion Sims. It would appear that the late Professor Nélatou was the first surgeon who introduced this practice. The author also thinks that the inverted position tends to drive from the lungs and trachea pent-up vapors of chloroform, which were increasing the asphyxia.—*London Lancet.*

---

*A Little-Studied Cause of Hoarseness.*—The hoarseness to which many public speakers become subject is generally regarded as a symptom of some sort of laryngitis. Dr. H. Welsch, in the Bavarian *Intelligentzblatt*, points out, however, another cause which deserves to be borne in mind in treating the generally obstinate cases, and that is, that the defect of the voice occasionally arises from *partial paralysis of the crico-thyroidei* muscles. They are thus disabled from drawing down the thyroid cartilage, and the extension of the vocal cords requisite to distinct phonation is impossible. The glottis assumes a sort of funnel shape. The treatment he recommends is tincture of iodine externally, and faradization of the impaired muscles.—*Medical and Surgical Reporter.*

---

*Treatment of Salivation by Atropia.*—The patient, a woman of sixty-eight years, had two attacks of apoplexy followed by hemiplegia of the left side. On being admited into Dr. Ebstein's wards [Breslau Hospital] profuse salivation was observed. Acording to the patient it had begun a month previously. Atropia was administered internally without any effect. On the dose being increased, the quantity of saliva diminished. Atropia [the sulphate] was then injected hypodermically, and after seven minutes the salivation was stopped. On doubling the dose the secretion was arrested for twelve hours. Dr. Ebstein explains the action of the drug through its influence on the permanent irritation of the secretory fibres of the salivary glands.—*Lancet.*

---

*Cancer Cured by Faith.*—Dr. Charles Cullis, of Boston, reports the following: " A lady came to me with a cancer in the cheek, which had attained the size of a filbert. It had a very red and angry appearance. After prayer for her healing, she went into the country, when some one remarked, ' E. thinks that faith will cure her, but this is something that will have to be burned out or cut out.' Her friends tried to induce the use of various applications, all of which she firmly refused. She returned home in *eight weeks*, entirely cured. The friends acknowledge that ' faith did do good once.' ".—*Medical Investigator.*

---

*Remedy for Chronic Hoarseness.*—An eminent physician of Philadelphia contributes the following: In chronic hoarseness arising from thickening of the vocal chords and adjacent membrane the ammoniated tincture of guaiacum is often a very efficacious remedy. It may be appropriately mixed with equal parts of the syrup of senega, and a teaspoonful of the mixture given two or three times a day.—*Druggists' Circular.*

---

*Administration of Ether.*—At the Bellvue Hospital, New York, the administration of potass. bromid. gr. xxx, previous, and the same amount immediately following, or as soon as the patient can conveniently swallow, after the administration of sulphuric ether for the purpose of producing anæsthesia, is now regularly resorted to. The effect is to prevent the vomiting which so commonly follows the use of the anæsthetic.—*Medical Record.*

---

*The Areca or Betel Nut.*—This nut is the best known as a dentifrice, and masticatory, very popular with the Orientals. In Bombay it is said to be used also with good effect as an anthelmintic.

The natives pick it off the tree, and grate it on an ordinary nutmeg grater. About a teaspoonful is administered, after the patient has fasted twelve or fourteen hours, either made up into a bolus with ghee ( clarified butter ) or floating on milk, the latter being the favorite method. It generally acts ( without any other medicine being given ) in about an hour after administration, and is efficacious for round as well as tape worms. It is used both for the human subject and dogs, in the same manner and dose.—*Med. and Surg. Reporter.*

---

*Laughter as a Medicine.*—A short time since, two individuals were lying in one room, very sick, one with brain fever, and the other with an aggravated case of the mumps. They were so low that watchers were needed every night, and it was thought doubtful if the one sick of fever could recover. A gentleman was engaged to watch over night, his duty being to wake the nurse whenever it became necessary to administer medicine. In the course of the night both watcher and nurse fell asleep. The man with the mumps lay watching the clock, and saw that it was time to give the fever patient his potion. He was unable to speak aloud, or to move any portion of his body except his arms, but, seizing a pillow, he managed to strike the watcher in the face with it Thus suddenly awakened, the watcher sprang from his seat, falling to the floor, and awakened both the nurse and the fever patient. The incident struck the sick man as very ludicrous, and they laughed heartily at it for some fifteen or twenty minutes. When the doctor came in the morning he found his patient vastly improved ; said he never knew so sudden a turn for the better, and now both up and well. Who says laughter is not the best of medicines? And this reminds the writer of another case. A gentleman was suffering from an ulceration in the throat, which at length became so swollen that his life was despaired of. His household came to his bedside to bid him farewell. Each individual shook hands with the dying man, and then went away weeping. Last of all came a pet ape, and shaking the man's hand, went away also with its hands over its eyes. It was so ludicrous a sight that the patient was forced to laugh, and laughed so heartily that the ulcer broke, and his life was saved.—*The Sanitarian for May.*

*Hygiene of Dwellings.*—Remarkable testimony as to the permeability of the ground, and of the foundations of our houses, has been given by gas emanations into houses which had no gas laid on I know cases where persons were poisoned and killed by gas which had to travel for twenty feet under the street, and then through the foundations, cellar-vaults, and flooring of the ground-floor rooms. As these kinds of accidents happened only in winter, they have been brought forward as a proof that the frozen soil did not allow the gas to escape straight upwards but drove it into the house. I have told you already why I take frozen soil to be not more air-tight than when not frozen.

In such cases the penetration of gas into the houses is facilitated by the current in the ground-air caused by the house.

The house being warmer inside than the external air, acts like a heated chimney on its surroundings, and chiefly on the ground upon which it stands and the air therein, which we will call the ground-air.

The movement of gas through the ground into the house may give no warning that the ground-air is in continual intercourse with our houses, and may become the introducer of many kinds of lodgers. These lodgers may either be found out, or cause injury at once, like gas; or they may, without betraying their presence in any way, become enemies, or associate themselves with other injurious elements, and increase their activity. The evil resulting therefrom continues till the store of these creatures of the ground-air is consumed. Our senses may remain unaware of noxious things which we take in, in one shape or another, through air, water, or food.—*Pettenkoffer, Sanitarian for May.*

---

*Guarana for the Cure of Sick Headache.*—By T. P. Caillout, of Lockport, La. MM. Trosseaux and Pidoux, in their *Traitr de Therapeutique et de Matiere Medical,* describe the mode of using the guarana powder in sick headache, and since the year 1861 I have used it with complete success in all patients who have applied to me for relief for the above mentioned malady. I did not use the extract recommended by Prof. Trosseau, but gave the powder in doses of ten to fifteen grains, repeated every two hours, and the patients rarely took three doses before they were relieved. A ten-grain dose taken immediately before the attack always prevented it.

Some of my patients, who suffered every month from sick-headache, were entirely rid of their sufferings for several months, and some for one and two years, after using one or two boxes of the powder, each box containing a dozen packages of ten grains each. · I recommended my patients to take a dose (of the guarana) as soon as they felt any symptoms of an attack coming on; during the attack a dose every two or three hours until relieved. *Med. News and Library.*

